# Temporal transcriptomic profiling reveals dynamic changes in gene expression of *Xenopus* animal cap upon activin treatment

**DOI:** 10.1038/s41598-021-93524-x

**Published:** 2021-07-15

**Authors:** Yumeko Satou-Kobayashi, Jun-Dal Kim, Akiyoshi Fukamizu, Makoto Asashima

**Affiliations:** 1 Strategic Innovation and Research Center, Teikyo University, 2-11-1 Kaga, Itabashi-ku, Tokyo, 173-8605 Japan; 2 Advanced Comprehensive Research Organization, Teikyo University, 2-11-1 Kaga, Itabashi-ku, Tokyo, 173-8605 Japan; 3 Life Science Center for Survival Dynamics, Tsukuba Advanced Research Alliance (TARA), University of Tsukuba, 1-1-1, Tsukuba, Tennoudai Ibaraki 305-8577 Japan; 4 Division of Complex Bioscience Research, Department of Research and Development, Institute of National Medicine, University of Toyama, 2630 Sugitani, Toyama, 930-0194 Japan

**Keywords:** Developmental biology, Stem cells

## Abstract

Activin, a member of the transforming growth factor-β (TGF-β) superfamily of proteins, induces various tissues from the amphibian presumptive ectoderm, called animal cap explants (ACs) in vitro. However, it remains unclear how and to what extent the resulting cells recapitulate in vivo development. To comprehensively understand whether the molecular dynamics during activin-induced ACs differentiation reflect the normal development, we performed time-course transcriptome profiling of *Xenopus* ACs treated with 50 ng/mL of activin A, which predominantly induced dorsal mesoderm. The number of differentially expressed genes (DEGs) in response to activin A increased over time, and totally 9857 upregulated and 6663 downregulated DEGs were detected. 1861 common upregulated DEGs among all Post_activin samples included several Spemann’s organizer genes. In addition, the temporal transcriptomes were clearly classified into four distinct groups in correspondence with specific features, reflecting stepwise differentiation into mesoderm derivatives, and a decline in the regulation of nuclear envelop and golgi. From the set of early responsive genes, we also identified *the suppressor of cytokine signaling 3* (*socs3*) as a novel activin A-inducible gene. Our transcriptome data provide a framework to elucidate the transcriptional dynamics of activin-driven AC differentiation, reflecting the molecular characteristics of early normal embryogenesis.

## Introduction

Embryogenesis is precisely regulated by various inductive interactions leading to tissue- or organ-specific gene expression programs and generation of cell fate diversity. In amphibian development, mesoderm induction is one of the primary embryonic interactions. During this process, a signal released from vegetal cells (the presumptive endoderm) converts the ectoderm into the mesodermal fate, establishing the three germ layers. Since the discovery of mesoderm formation depending on inductive events, the identification of mesoderm-inducing substances and their functions have been investigated^1,2^.

Activin has been identified as a potential mesoderm inducing factor based on its capacity to induce into the mesodermal and endodermal lineage in vitro. Following treatment with activin in combination with other morphogenic proteins such as bone morphogenetic proteins (BMPs) and fibroblast growth factors (FGFs), embryonic stem cells are differentiated into mesoderm or definitive endoderm^3–6^. In amphibian experiments, animal cap explants (ACs) isolated from the animal pole region of blastulae differentiate autonomously into atypical epidermis, but they can differentiate into various endodermal and mesodermal tissues upon activin treatment^7–10^. As for mesoderm induction, activin at higher concentrations induces dorsal types such as muscle and notochord, and at lower concentrations induces ventral type in ACs^10^. This concentration-dependent tissue induction is due to the behavior of activin as a morphogen in dorsoventral patterning of mesoderm^11–13^. One of the fascinating features of activin-treated ACs is their ability to mimic the activity of the signaling center, Spemann’s organizer. After the transplantation of activin-treated ACs into the blastocoel of another embryo, a secondary axis is induced, similar to transplantation experiments of the organizer dissected from the embryo^14^. Thus, activin-treated ACs can mimic the organizer’s activity, but it remains unclear how and to what extent ACs differentiation in vitro recapitulates the organizer formation in vivo.

The recently developed deep RNA sequencing (RNA-seq) provides transcriptional information with a high resolution and sensitivity. This technique has been widely applied to cultured cells, model organisms, as well as to gastruloids and organoids of developmental models^15–18^. In *Xenopus* research, RNA-seq protocols and pipelines for data analysis have been developed in recent years, and several reports using this approach have been published^19–22^. In particular, transcriptome analysis of the dorsal marginal zones in *Xenopus* gastrula that contain dorsal mesoderm populations has been useful for understanding mesoderm developmental in vivo^23,24^.

In the present study, we performed a time-course transcriptome profile of *Xenopus* ACs treated with 50 ng/mL of activin A, which was known to predominantly induce dorsal mesoderm^10^. We identified stepwise variation in gene expression, as well as the enriched biological processes, during activin A-driven AC differentiation. From the results, we detected several known organizer genes, and captured the transition from early unrestricted gene expression to mesoderm lineage commitment later. Furthermore, we found that activin A treatment resulted in the potent activation of the early-responsive genes, but the gene expression patterns of late-responsive genes in ACs were similar to those in the gastrula embryos^25^. We further reported s*ocs3* as a novel activin A-inducible gene and inferred the *socs3*-associated genes. Thus, our data provide information of the temporal dynamics in gene expression during activin A-induced ACs differentiation, and further support the usefulness of the in vitro ACs assays for developmental studies.

## Results

### Validation of mesoderm-inducing activity by activin A treatment

We first validated the effective concentrations of activin A for the induction of dorsal mesoderm genes by semiquantitative RT-PCR. As shown in Supplementary Fig. S1, 50 ng/mL activin A predominantly induced dorsal mesoderm genes, compared with 10 ng/mL activin A, which is consistent with previous findings that higher concentrations of activin induce dorsal mesoderm^11–13^. We then validated the ability of activin A at 50 ng/mL concentration to induce dorsal mesoderm at later time points. As shown in Supplementary Fig. S2A, activin A-treated ACs exhibited extensive elongation, which is reportedly caused by the differentiation of notochord and somite^10^. Furthermore, we found that the expression of the notochord marker, *chrd*, and the somite markers, *myf5*, *actc1* and *myogenin* were induced after 48 h of activin A treatment (Supplementary Fig. S2B). These data indicate that 50 ng/mL activin A is the optimal concentration for ACs differentiation into dorsal mesoderm.

### Transcriptomic changes in activin A-treated ACs

**Figure 1:**
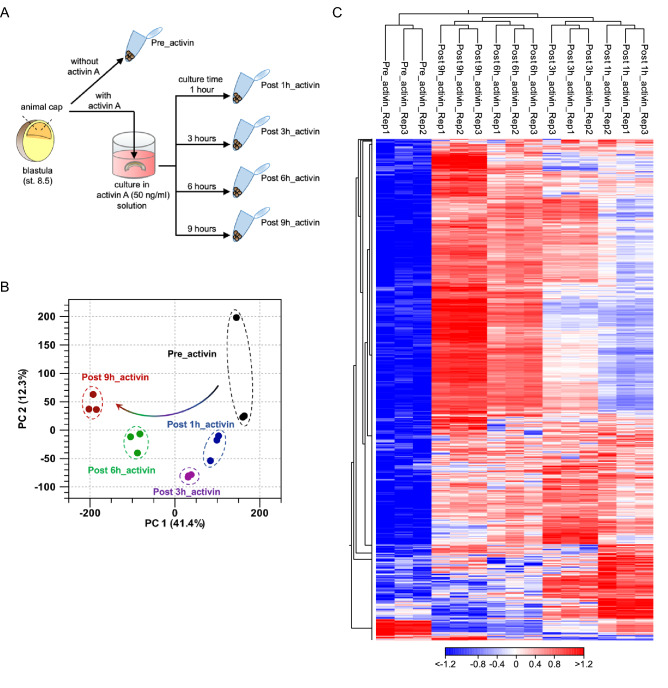
Transcriptomic relationships among AC samples. (**A**) Overview of the sample preparation for a time-course RNA-seq. ACs were collected without activin A treatment (Pre_activin), and after activin A treatment for 1, 3, 6 and 9 h (Post 1h_activin, Post 3h_activin, Post 6h_activin, and Post 9h_activin). (**B**) Principal component analysis (PCA) of ACs. The first two principal components PC1 and PC2 represented 53.7% of the total variance. Each dot denotes a single biological replicate, and dashed circles represent three replicates for each individual sample. Black dot, Pre_activin; blue dot, Post 1h_activin; purple dot, Post 3h_activin; green dot, Post 6h_activin; red dot, Post 9h_activin. (**C**) Hierarchical clustering of the expression profiles between Pre_activin and Post_activin groups. Individual samples are shown in columns, and genes in rows. The upper axis shows the clusters of samples, and the left vertical axis shows clusters of genes. The heatmap represents relative expression (red, high; white, intermediate; blue, low expression). Rep1-3; the biological replicate 1–3. The heatmap was visualized using the CLC Genomic Workbench software 12.0 (QIAGEN).

To comprehensively understand the temporal gene expression during activin A-induced ACs differentiation, we collected ACs immediately after dissection from blastula (Pre_activin; sibling embryos reached stages 8.5–9), and ACs after the cultivation in activin A solution for 1, 3, 6 and 9 h (Post 1h_activin; stage 9.5, Post 3h_activin; stages 10–10.25, Post 6h_activin; stages 10.5–11, and Post 9h_activin; stage 11.5, respectively) (Fig. 1A), and performed a time-course RNA-seq to analyze the transcriptome. To assess the dynamics of gene expression variability following activin A treatment, we performed principal component analysis (PCA) and hierarchical clustering analysis. PCA using all samples showed that there was a clear distinction between the same groups at five time points, and the transcriptomic changes in ACs from Pre_activin to Post 9h_activin (Fig. 1B). Moreover, in hierarchical clustering analysis, we found that Pre_activin samples were distinguished from Post_activin groups (Post 1 h, 3 h, 6 h, and 9h_activin), which were further divided into two subgroups, short-term (Post 1 h and 3h_activin) and long-term activin A treatment (Post 6 h and 9h_activin) (Fig. 1C). These data suggest that the gene expression of ACs is significantly changed after activin A treatment in a temporal manner.

We then validated the accuracy of gene expression patterns observed by RNA-seq with a semiquantitative RT-PCR. We focused on *ccnb1* as a representative downregulated gene, and *eomesodermin* (*eomes*), *lhx1*, *myf5*, *t*, *goosecoid* (*gsc*), *otx2*, *chordin* (*chrd*), *wnt8a* and *cerberus 1* (*cer1*) as induced genes upon activin A treatment^26–31^. The temporal expression patterns of these representative genes observed by RT-PCR were similar to the data from RNA-seq, with minor differences (compare Supplementary Fig. S3B to S3C, and blue lines in Supplementary Fig. S1 to Supplementary Fig. S4).

### Differentially expressed genes profiles between Pre_activin and Post_activin samples

**Figure 2:**
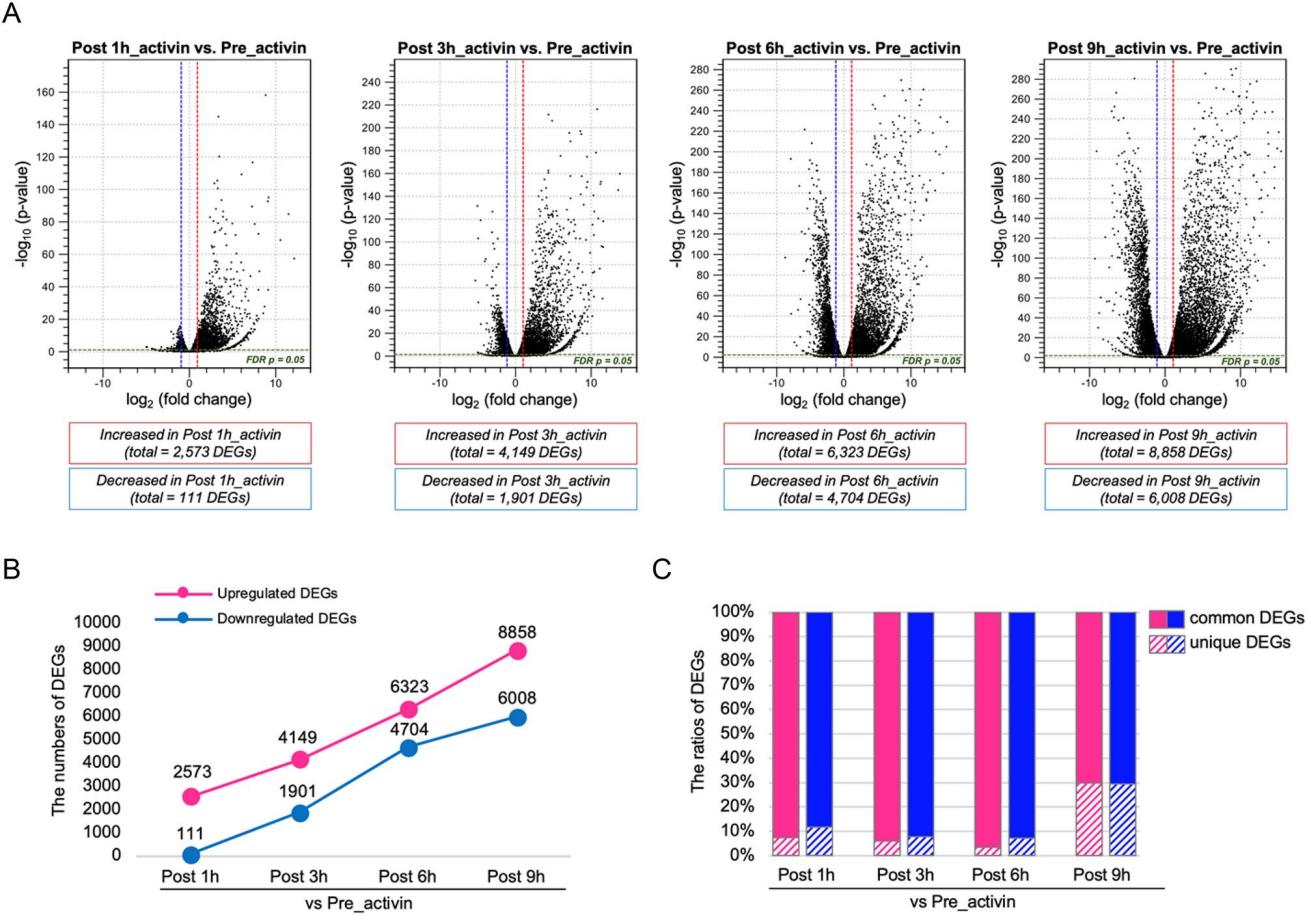
Differentially expressed genes (DEGs) between Pre_activin and each Post_activin sample. DEGs were identified in the comparison of gene expression between Pre_activin and each Post_activin sample based on log2 FC ≥ 2 or ≤  − 2 with FDR at p < 0.05. (**A**) Volcano plots representing DEGs in each comparison. Each dot represents individual DEG. Dotted vertical lines, log2 FC ≥ 2 or ≤  − 2; dotted horizontal line, the significance cut-off (p < 0.05). (**B**) Line graph showing the total number of upregulated and downregulated DEGs in each comparison. (**C**) Bar chart showing the ratio of common DEGs among different Post_activin samples and that of unique DEGs in each Post_activin sample. Common DEGs and unique DEGs are represented by solid or stripe pattern fill, respectively. (**B**,**C**) Magenta, upregulated DEGs; blue, downregulated DEGs.

We next analyzed the statistically significant differentially expressed genes (DEGs) between activin A-treated and -untreated samples. Based on a false discovery rate (FDR) of less than 0.05 and a log2 fold change (FC) ≥ 2 or ≤  − 2, we extracted DEGs in Post_activin samples compared to the Pre_activin sample as a control. The full list of DEGs is shown in Supplementary Table S1, and the top five upregulated and downregulated DEGs in each comparison are shown in Table 1. As shown in Fig. 2A, volcano plots showed that 2573, 4149, 6323, and 8858 genes were upregulated, and 111, 1901, 4704 and 6008 genes were downregulated in the Post 1 h, 3 h, 6 h, and 9h_activin samples, respectively. Thus, the number of DEGs gradually increased over time after treatment with activin A, but more upregulated DEGs were detected than downregulated DEGs across all time points (Fig. 2B). As shown in Fig. 2C, the ratio of unique DEGs (non-overlapping DEGs among different Post_activin samples) in Post 9h_activin sample was higher than other Post_activin samples, implying that time point- or context-specific gene expression actively occurs at least 9 h after treatment with activin A.

**Table 1:** List of top five upregulated and downregulated DEGs.

	Post 1h_activin (vs Pre_activin)	Post 3h_activin (vs Pre_activin)	Post 6h_activin (vs Pre_activin)	Post 9h_activin (vs Pre_activin)
Upregulated	*socs3.L* *socs3.S* *foxa4.L* Undescribed-1* *foxa4.S*	*foxa4.L* *foxa4.S* *pcdh8.2.S* *unc93a.L* *socs3.L*	*pcdh8.2.L* *foxa4.L* *foxa4.S* *pcdh8.2.S* *chrd.2.L*	*foxa4.L* *chrd.2.L* *pcdh8.2.L* *pnhd.S* *foxa4.S*
Downregulated	*pdp2.L* Undescribed-2* *plekhg7.L* Undescribed-3* *spef1.S*	Undescribed-4* *cpeb1.L* *nkx3-3.L* *cpeb1.S* Undescribed-5*	Undescribed-4* *ccnb5.L* Undescribed-6* Undescribed-7* Undescribed-8*	Undescribed-4* *ccnb5.L* *msmo1.L* Undescribed-6* Undescribed-5*
Undescribed-1*, Xelaev18038693m.g; Undescribed-2*, Xelaev18005995m.g; Undescribed-3*, loc100487103.1; Undescribed-4*, Xelaev18036268m.g; Undescribed-5*, Xelaev18004162m.g; Undescribed-5*, Xelaev18004162m.g; Undescribed-6*, Xelaev18004163m.g; Undescribed-7*, loc100494194.L; Undescribed-8*, Xelaev18008393m.g				

DEGs were identified in the comparison of each Post_activin vs. Pre_activin sample. Sequences that did not show the significant hits or annotations (listed as model IDs starting with “loc” and “Xelaev”) were represented as undescribed 1–8. *X. laevis* is an allotetraploid, and contains two sets of genes, L and S genes.

**Figure 3:**
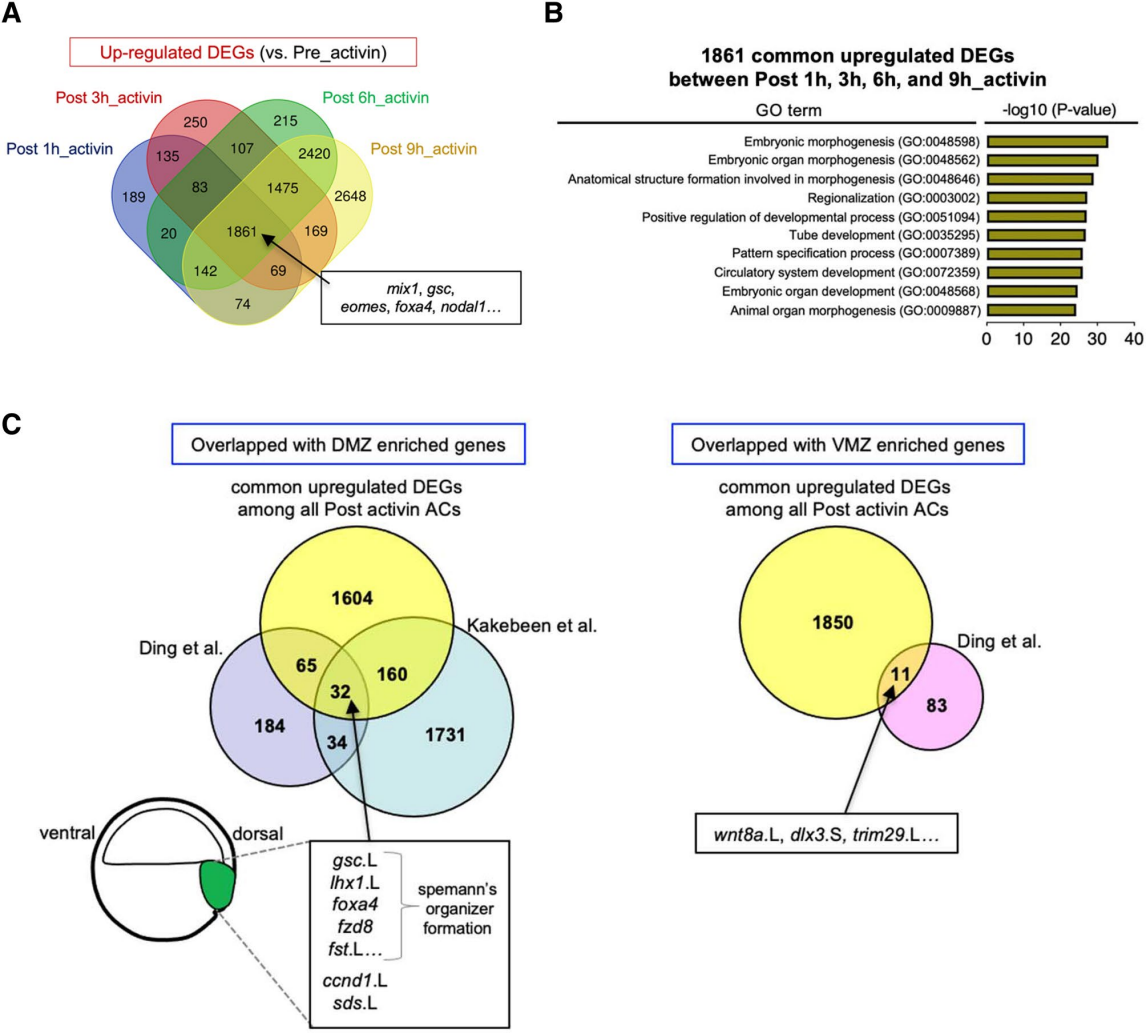
Common upregulated DEGs among Post_activin samples. (**A**) Venn diagrams showing overlap among upregulated DEGs. The numbers of common and unique upregulated DEGs are displayed in colored ellipses. Known activin responsive genes are listed as indicated. (**B**) Gene ontology (GO) enrichment analysis of 1861 common upregulated DEGs between Post 1 h, 3 h, 6 h, and 9h_activin. The top ten enriched GO terms in biological processes, and the -log10 (P-value) of the significant GO terms are displayed in order of significance from the bottom of the list. (**C**) Left Venn diagram showing the comparison of DMZ-enriched genes at stage 10.5 (Ding et al.), those at stage 11 (Kakebeen et al.) and common upregulated DEGs among all Post_activin samples. The representatives of overlapping genes among three studies are indicated. Right Venn diagram showing the comparison of VMZ-enriched genes at stage 10.5 (Ding et al.) and common upregulated DEGs among all Post_activin samples. The numbers of common and unique genes in independent studies are displayed in colored circles.

According to the data set of DEGs shown in the Venn diagrams, 1861 genes including activin-responsive targets, *mix1*, *gsc*, *eomes*, *foxa4* and *nodal1*^32–36^, were commonly upregulated (Fig. 3A and Supplementary Table S2), and only 70 genes were commonly downregulated among all Post_activin samples (Supplementary Fig. S5 and Supplementary Table S2). To characterize the functional features of DEGs in ACs following activin A treatment, we performed gene ontology (GO) functional annotation of common upregulated DEGs. Common upregulated DEGs among all Post_activin samples were associated with animal organ morphogenesis (GO:0009887) and embryonic organ development (GO:0048568) (Fig. 3B).

To gain the understanding of genes induced by activin A, we compared the DEGs in response to activin A with the set of genes enriched in the marginal zone of the *Xenopus* gastrula. The transcriptome datasets of dorsal marginal zone (DMZ)- and ventral marginal zone (VMZ)-enriched genes were obtained from different groups^23,24^, and compared with common upregulated DEGs among all Post_activin samples. As shown in Fig. 3C, we detected the overlaps of DMZ-enriched genes including *gsc*.L, *lhx1*.L, *foxa4*, *fzd8*, and *follistatin* (*fst*.L) that are involved in Spemann’s organizer formation, as well as *ccnd1*.L encoding cyclin D1, and *sds*.L encoding serine dehydratase (left). In addition, a few VMZ-enriched genes including *wnt8a*.L, *dlx3*.S, and *trim29*.L overlapped with upregulated DEGs in response to activin A (Fig. 3C right).

### Classification of genes that exhibited distinct expression pattern

**Figure 4:**
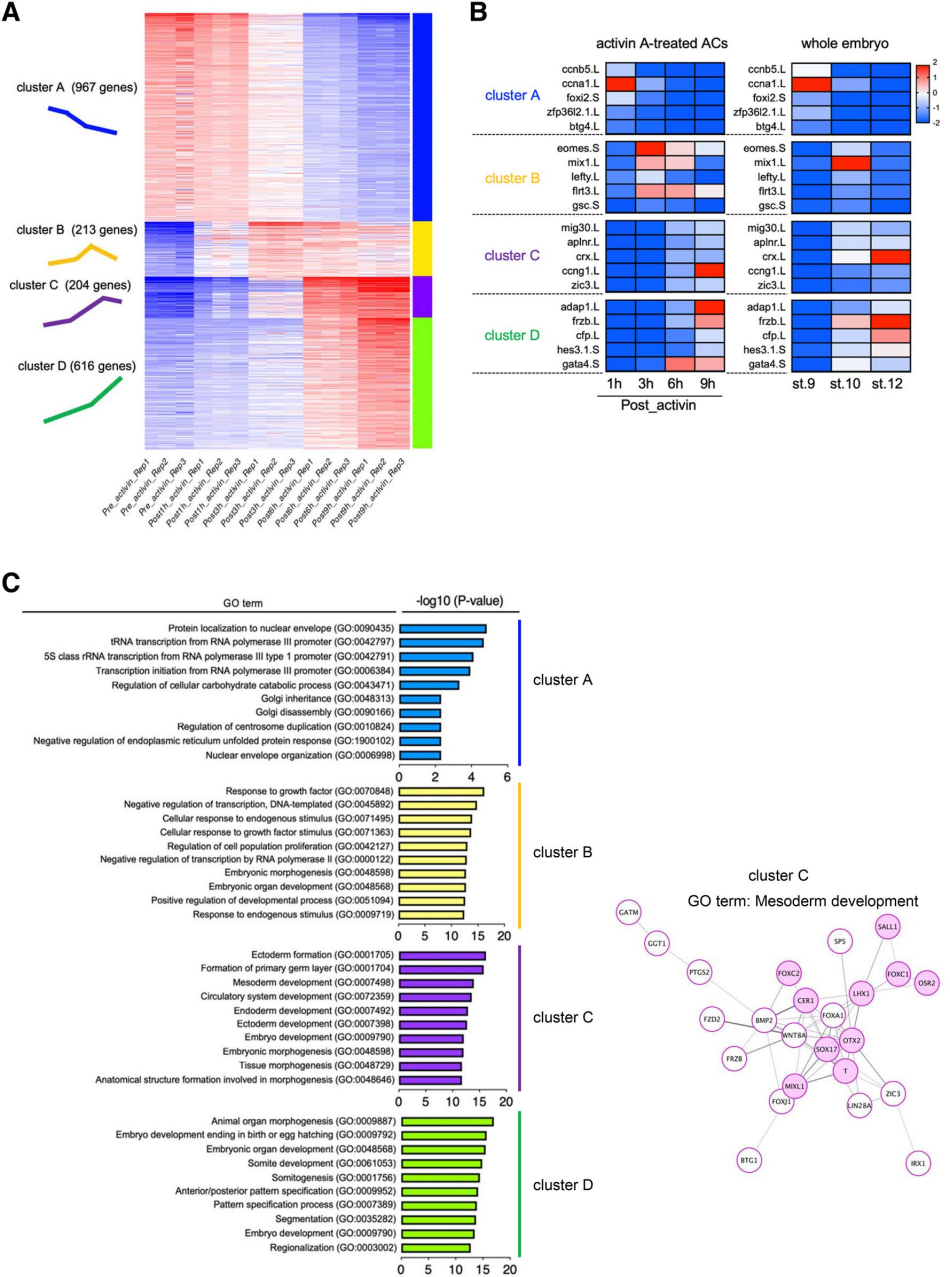
Clustering of gene expression trajectories. (**A**) K-means clustering of 2000 variable genes following activin A treatment. Genes were classified into four clusters based on similar expression patterns. Individual samples are displayed on the vertical axis, and genes on the horizontal axis. The colors in heatmap indicates relative expression of each tile (red, high; white, intermediate; blue, low expression). The heatmap was visualized using the iDEP.93 online tools (http://bioinformatics.sdstate.edu/idep/). The number of genes and schematic diagrams representing specific expression patterns in each cluster are shown on the left. (**B**) Heatmaps showing the gene expression of five representatives in each cluster. The expression of genes observed in activin A-treated ACs (left), and that in whole gastrula embryos (stages 9, 10 and 12) (right). The transcriptomes of the whole embryos were obtained from “Session et al.” dataset. The color scale bar is shown at the top right corner of the figure. The heatmap represents relative gene expression (red, high; white, intermediate; blue, low expression). The heatmaps were visualized using the CLC Genomic Workbench software 12.0 (QIAGEN). (**C**) GO enrichment analysis of genes in different clusters and the genes of cluster C assigned in the mesoderm development GO term. (left) The top ten enriched GO terms in biological processes, and the -log10 (P-value) of the significant GO terms are displayed in order of significance from the bottom of the list. Results of GO analysis in each cluster are indicated by different colors: blue, cluster A; yellow, cluster B; purple, cluster C; green, cluster D. (right) The predicted protein–protein interaction (PPI) network for genes in cluster C. Nodes associated with mesoderm development represent as pink circles, and other nodes as white circles. Edges are as grey lines. The thickness of connected lines indicates the strength of the interaction.

To identify the set of genes with similar temporal expression patterns, we performed k-means clustering for 2000 genes, which were variably expressed after activin A treatment. We identified four clusters with distinct expression patterns (Fig. 4A and Supplementary Table S3). Cluster A (967 genes) contained a set of genes that was downregulated over time after activin A treatment. Clusters B, C, and D contained genes that were upregulated after activin A treatment, but there was little difference in gene expression patterns. Cluster B (213 genes) contained genes whose expression was significantly upregulated at 1 or 3 h and subsequently downregulated at later time points. Cluster C (204 genes) contained genes whose expression was markedly from 3 or 6 h, and slightly downregulated but maintained at higher expression levels at later time points. Cluster D (616 genes) contained genes that were drastically upregulated at 6 or 9 h.

To compare activin A-induced ACs differentiation in vitro with in vivo development, we presented the temporal gene expression of five representatives of each cluster in activin A-treated ACs and that in whole gastrula embryos. The transcriptome dataset of the whole gastrula embryos was obtained from published RNA-seq data^25^. As shown in Fig. 4B, the downregulation of genes in cluster A was observed in both cultured ACs and whole embryos. Conversely, the expression of genes in cluster B was actively induced in ACs at the early time point (in Post 3 h and 6h_activin ACs), but these genes were modestly induced in whole embryos (Fig. 4B). The expression of genes in cluster C and cluster D increased in ACs later (in Post 9h_activin ACs), and similar temporal changes in gene expression were observed in whole embryos (Fig. 4B).

To further investigate the functional diversity of DEGs over time after activin A treatment, we next performed functional enrichment analysis on each cluster. The top ten GO terms in each cluster are listed in Supplementary Table S4. GO enrichment analysis of biological processes showed that genes in cluster A were associated with nuclear envelop organization (GO:0006998), regulation of endoplasmic reticulum (GO:1900102) and golgi (GO:0090166, GO:0048313) (Fig. 4C and Supplementary Table S4). Notably, genes in cluster B were associated with growth factor stimulus (GO:009719, GO:0071363, GO:0071495, GO:0070848), in cluster C were associated with primary germ layer formation (GO:0009798, GO:0007492, GO:0007498, GO:0001704, GO:0001705), and those in cluster D were associated with regionalization (GO:0003002), pattern specification (GO:0007389, GO:0009952), and somite formation (GO:0001756, GO:0061053) (Fig. 4C and Supplementary Table S4). In addition, the predicted protein–protein interaction (PPI) network for genes in cluster C showed that some genes, encoding transcription factors such as OTX2, SOX17, T, and LHX1, were assigned in the mesoderm development GO term (GO: 0007498) (Fig. 4C pink circles, Supplemental Table S5).

### Upregulation of *socs3* in response to activin A

**Figure 5:**
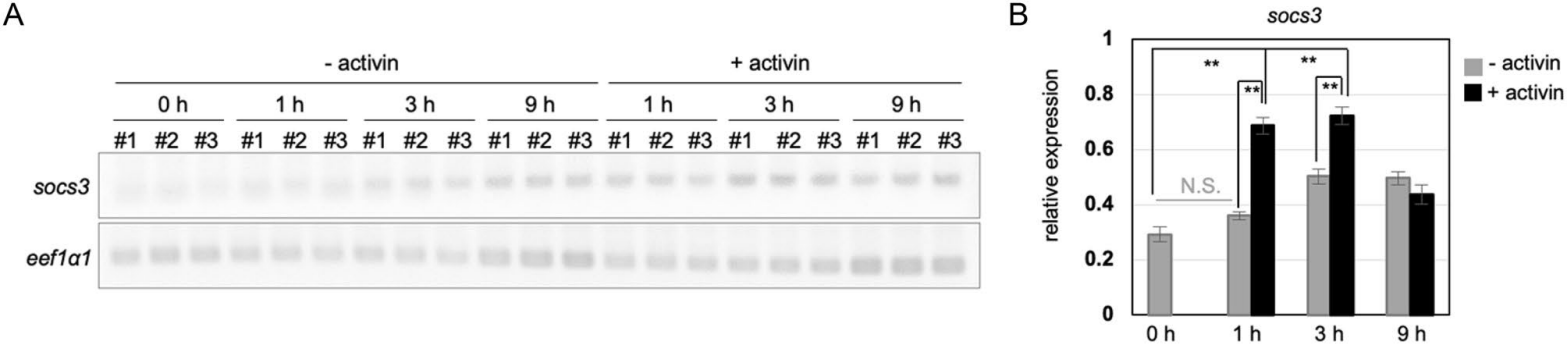
Upregulation of *socs3* in ACs following activin A treatment. Semiquantitative RT-PCR of the expression of *socs3* in ACs. Dissected ACs were cultured with or without of activin A. (**A**) Electrophoresis gel of RT-PCR products. #1–3 indicate the three biological replicates. The upper panel shows the expression of *socs3* and the lower panel shows the expression of *eef1a1*, which was used as the loading control. Full size images of each gel are presented in Supplementary Figure S10. For (**B**), the densitometry for *socs3* expression. Error bars indicate s.e.m. (n = 3). **p < 0.01, *p*-value was calculated using Student’s t-test after the one-way analysis of variance. N.S., not significant. (**A**,**B**) Cultivation times are indicated as follows: 0 h, 0 h (the absence of cultivation); 1 h, 1 h; 3 h, 3 h; 9 h, 9 h.

The most significantly upregulated DEGs in activin A-treated ACs at the early time point was *socs3* (Table 1, Post 1h_activin). Therefore, we focused on *socs3* as a possible activin-inducible gene. *socs3* is reportedly involved in cytokine signaling, and induced in response to wound healing after epithelium trauma^37^. To determine whether *socs3* is upregulated in response to activin A treatment, we examined the temporal expression of *socs3* in ACs with or without activin A treatment using semiquantitative RT-PCR. As shown in Fig. 5, *socs3* was slightly expressed in ACs before the cultivation (− activin, 0 h), but significantly upregulated after 1 and 3 h of activin A treatment (+ activin, 1 h and 3 h), compared with in the absence of activin A treatment at the same time points (− activin, 1 h and 3 h). These data suggest that *socs3* was maternally expressed or induced in response to the traumatic dissection from blastulae, but further induced after activin A treatment. Thus, we validated that RNA-seq can be used to identify novel activin-inducible genes in ACs. To predict the *socs3*-associated genes in activin A-treated ACs, we inferred a gene network using temporal gene expression profiles of upregulated DEGs in Post 3h_activin samples. In the inferred network, we found many potential *socs3*.S-associated genes including *pou5f3.2*.S, *gata4*.S, and *map3k1*.S (Supplementary Fig. S6, Supplementary Table S6).

## Discussion

In this study, we captured the transcriptional features of ACs that had the potential to develop into dorsal mesoderm. Although the transcriptional organization in activin A-treated ACs did not completely correspond to DMZ-expressing genes, activin A-treated ACs expressed a variety of dorsal mesoderm genes that are involved in Spemann’s organizer formation. We also found that temporal gene expression patterns were similar between activin A-treated ACs and whole embryos, with some notable differences. We also provided the data on expression of *socs3* as a potential activin-inducible gene, which was identified from our RNA-seq dataset.

In the present study, we confirmed that *mix1*, *gsc*, *eomes*, *foxa4* and *nodal1* were rapidly upregulated, and *lhx1*, *otx2*, *chrd*, *cer1, wnt8*, and *ventx* were also upregulated following activin A treatment (Supplementary Tables S1 and S2), as previously reported^27,29–36,38^. In *Xenopus* gastrula embryos, these genes are expressed in the Spemann’s organizer and the ventral mesoderm (Supplementary Fig. S7), and play important roles in the endoderm and mesoderm formation^39^. Although activin signaling acts as a morphogen and induces both ventral and dorsal mesoderm^11–13^, the upregulated DEGs following 50 ng/mL activin A treatment in ACs overlapped with DMZ-enriched genes more than VMZ-enriched genes (Fig. 3C). These results suggest that this concentration of activin A was sufficient to induce and maintain the dorsal mesoderm.

Activin signaling regulates other signaling pathways for mesoderm development in *Xenopus*. For example, activin reportedly induces the expression of *fgf3/*8, and also induces the negative regulator of FGF signaling, *dusp1*, for the dorsoventral patterning of mesoderm^40^. Furthermore, activin signaling reportedly activates the expression of downstream genes of p53 signaling, *p21waf1* and *pal-1,* and the mesendoderm gene, *mix.2*, through the cooperative action with p53^41–43^. Consistent with the findings of previous reports, we confirmed the induction of these genes (Supplementary Tables S1 and S2), and detected the relevance of upregulated DEGs to FGF and p53 signaling from the GO and Kyoto Encyclopedia of Genes and Genomes (KEGG) pathway analysis (Supplementary Table S4). Thus, activin signaling can modulate other signaling pathways for establishment of mesoderm in ACs. Notably, the expression of *siamois* (*sia1* and *sia2*) that acts downstream of Wnt signaling and is essential for the formation of the Spemann’s organizer^44^ was not significantly induced in activin A-treated ACs (Supplementary Table S7). Furthermore, all previously reported DMZ-enriched genes did not overlap with upregulated DEGs in response to activin A (Fig. 3C). These differences in gene expression between differentiated ACs and embryonic development are possibly due to the limited effects of activin A at this concentration in ACs, or due to other signaling derived from the neighboring cells in the embryo, but are not in ACs.

In this study, we classified upregulated genes whose expression reached the peak at early (Post 1 h and 3 h), intermediate (Post 3 h and 6 h), and later time points (Post 6 h and 9 h) following activin A treatment (Fig. 4A; corresponding to cluster B, C and D). The expression of activin-early responsive genes (cluster B) was actively induced in ACs than in whole gastrula embryos (Fig. 4B). In addition, the temporal trends in expression of intermediate- or late-responsive genes (clusters C and D) in ACs were highly consistent with those observed in gastrulae, although there were some differences in transcription levels and temporal association (Fig. 4B). These data imply that the primary or direct effects of activin A on early-responsive genes result in the downstream gene expression in concordance with normal development. As for the activin-responsive transcription, several activin-responsive targets were identified from the experiments using the combinatorial treatment of activin and the protein synthesis inhibitor, cycloheximide^26,27,33^. In addition, genes associated with transcription factors of activin/nodal signaling, Smad2/3 and FoxhI, were determined by the chromatin immunoprecipitation sequencing (ChIP-seq) using *X. tropicalis* gastrulae^45,46^. We confirmed that several activin-responsive target genes and Smad2/3-FoxhI associated genes were upregulated in ACs at early time points after activin A treatment (Supplementary Table S3 and Supplementary Fig. S8). It suggests the possibility that the effects of activin A on direct target genes lead to the induction of indirect or secondary responsive genes, resulting in diverse gene expressions in ACs later.

Interestingly, numerous genes previously undescribed in mesoderm formation were observed. Especially, downregulated genes in ACs following activin A treatment have not been investigated well in former experiments, as compared to upregulated genes (Table 1). We first demonstrated that genes associated with the organization of nuclear envelop were downregulated in ACs following activin A treatment, for example, *ctdnep1* and *lemd3* (Fig. 4C and Supplementary Table S4). CTDNEP1, encoding a serine/threonine protein phosphatase, functions as a mediator of lipid composition in nuclear envelop, but it represses both TGF-β and BMP signal transduction^47–50^. Lemd3, encoding a nuclear membrane protein, also suppresses the TGF-β signal transduction through physical interaction with downstream transcription factors of TGF-β signaling, Smads^51,52^. These data suggest the possibility that the nuclear envelop is organized during ACs differentiation into the endodermal and mesodermal lineage, leading to reinforcement of the TGF- β signal activation. The role of downregulated genes associated with the regulation of golgi and endoplasmic reticulum and rRNA/tRNA transcription in early developmental processes remains to be elucidated (Fig. 4C), but our data may lead to the identification of novel molecular mechanisms in vivo development.

During the blastula stage of embryonic development, zygotic gene activation occurs, which is called the mid-blastula transition (MBT)^53,54^. It has been reported that the open chromatin accessibility for gene expression becomes higher after MBT (at least after stage 9) in *Xenopus* ACs^55^. Our transcriptome data using ACs dissected from blastula demonstrated the dynamic expression of various epigenetic regulators, which increase chromatin accessibility and lead to acceleration of gene transcription. For example, various *hdac* genes, encoding histone deacetylases, were downregulated (except for *hdac9.S*, *hdac10.L*, and *hdac7*), whereas *ep300* and *hat1*, encoding histone acetyltransferase, were upregulated in response to activin A treatment (Supplementary Tables S1 and S2). In general, histone acetylation targeted to lysines progressively increases transcriptional activation, whereas HDAC deacetylation activity represses transcription^56^. Our data demonstrating downregulation of *hdac* and upregulation of *ep300* and *hat* suggest the possibility that gene expression is activated by epigenetic mechanisms in ACs following activin A treatment. Another example of epigenetic regulators that alter the expression levels in response to activin A treatment is *dnmt1*, encoding DNA methyltransferase (Supplementary Tables S1 and S2). During normal development, Dnmt1 suppresses premature gene activation before MBT^57^, and the expression levels are downregulated from late blastula to early gastrula for zygotic gene activation^58^. Consistent with the data, we detected the downregulation of *dnmt1* in ACs upon activin A treatment (Supplementary Tables S1 and S2). Taken together, these data imply that the in vitro culture system using ACs treated with activin A recapitulates zygotic gene activation controlled by epigenetic mechanisms in normal development.

We also newly found that *socs3* was upregulated by activin A treatment (Table 1 and Fig. 5). *socs3* plays a role in skin inflammation^59^, and it has been reported to control the balance between stem cell pluripotency and differentiation into the primitive endoderm lineage via FGF-MAPK pathway^60,61^. In the inferred network, a pluripotency factor, *pou5f3.2*.S, an endoderm marker, *gata4*.S, and a downstream kinase of MAPK, *map3k1*.S, were obtained as potential *socs3*-associated genes (Supplementary Fig. S6). These previous findings with the predicted *socs3* subnetwork indicated that *socs3* is involved in the maintenance of ACs pluripotency and activin A-driven differentiation. Further research is needed to elucidate whether activin signaling regulates the pluripotent and differentiation states in *Xenopus* ACs via the modulation of *socs3* expression.

Taken together, our results demonstrating the developmental potency of ACs relied on activin A provide information required for early development. Our transcriptome in a mesendoderm-biased environment may serve as a resource for understanding the network during primary germ layer formation in combination with in vivo transcriptome datasets^23–25^ and previous GRN resources^62–65^. The in vitro ACs assay has some limitations, such as fewer cells and limiting growth factors at local levels, but it enables to understand features that could not be captured in complex processes within embryos.

## Methods

### Preparation of *Xenopus* embryos

The wildtype *X*. *laevis* strains were maintained in accordance with guidelines on animal housing^66^. All animal procedures were approved by the Animal Experimentation Committee of the Teikyo University (approval numbers: 18-020), and authors carried out all methods in our experiments in accordance with ARRIVE guidelines. Embryos were artificially fertilized, dejellied, and incubated in 0.1 × Steinberg’s solution^67^. Embryos were cultured until the blastula stages (stages 8.5–9) according to the normal table of Nieuwkoop and Faber^68^. The vitelline membrane was removed from embryos using forceps.

### ACs dissection

ACs were removed from blastula stage embryos (stages 8.5–9) using tungsten needles, as previously described^69^. Dissected ACs were cultured in activin A solution (50 ng/mL in 0.5 × Steinberg’s solution containing 0.1% BSA and 0.1 mg/mL kanamycin sulfate) at 18℃. As an activin A-untreated sample, ACs were collected immediately after dissection in the absence of activin A treatment. Authors carried out all methods in our experiments in accordance with relevant guidelines and regulations.

### Total RNA extraction, RNA library construction, and RNA-sequencing (RNA-seq)

Ten ACs per sample, in triplicate, were homogenized in ISOGEN (Nippon Gene). Total RNA was extracted according to the manufacturer's protocol and was purified using the RNeasy Mini kit (QIAGEN). For ethanol precipitation, the extracted RNA was mixed with ethanol and NaCl and then centrifuged at 4 °C for 30 min. After removal of supernatants, RNA pellet was washed with 80% ethanol, and was dried up and resuspended with RNase free water. RNA libraries were prepared for sequencing using standard Illumina protocols, and RNA sequencing were performed as described previously^70^. Reads were mapped to the X. laevis v9.2 genome (Xenbase, http://www.xenbase.org/entry/). All RNA-seq datasets are deposited in the NCBI GEO under accession number GSE153925.

### Identification of differentially expressed genes (DEGs)

For comparative analysis of gene expression between Pre_activin and each Post_activin sample, read counts were normalized by calculating the number of reads per kilobase per million (RPKM) for each transcript in individual samples using the CLC Genomics Workbench software version 12.0 (QIAGEN, CLC-LICENSE-1KPRB-BMN2G-24G14-CGCSY-6CQ28). The RPKM values for each gene are listed in Supplementary Table S7. DEGs between Pre_activin and Post_activin samples were filtered at cut-off of a false discovery rate (FDR) < 0.05 and a log2 fold change (FC) ≥ 2 or ≤  − 2. The comparison of distinct gene expression patterns was visualized in a principal component analysis (PCA), heatmaps, and volcano plots using the CLC Genomic Workbench software 12.0 (QIAGEN, CLC-LICENSE-1KPRB-BMN2G-24G14-CGCSY-6CQ28).

### Reverse transcription PCR (RT-PCR) assay

Equal amounts of total RNA extracted from each sample were subjected to RT-PCR as described previously^71^. PCR was performed using an EmeraldAmp PCR Master kit (Takara) using the following protocol: 28 cycles of 98 °C for 10 s, 55 °C for 30 s, and 72 °C for 30 s, followed by final elongation at 72 °C for 10 min. The sequences of the tested primers are listed in Supplementary Table S8. Electrophoresis with 1% agarose gel was carried out to verify the band size of the PCR products. For semiquantitative analysis, the band intensity of PCR products was quantified using Bio Image Intelligent Quantifier software (Bio Image Systems, Inc.) and the relative expression of the tested genes was calculated by dividing the band intensity of the tested genes with that of *eef1α1* as an internal control. Three pools in each sample were assayed for RT-PCR. Experiments were carried out at least twice, and a representative result is shown when similar results were obtained.

### Functional enrichment analysis and the inferred network

Gene ontology (GO) analysis and PPI network analysis were performed after conversion of the *X*. *laevis* genes to the human ortholog genes as previously described^72^. GO analysis and KEGG pathway analysis (Supplementary Table S4) of DEGs were performed using online ToppGene Suite (http://toppgene.cchmc.org). K-means clustering of DEGs was performed and visualized using iDEP.93 (integrated Differential Expression and Pathway analysis) online tools (http://bioinformatics.sdstate.edu/idep/)^73^. The PPI network was extracted from the human STRING network (version 11.0) and visualized using Cytoscape software (version 3.8.2, https://cytoscape.org). PPI functional enrichment was performed using String Enrichment plugin in Cytoscape^74^. The gene network was inferred from the temporal gene expression profiles (transformed gene expression in Pre_activin, Post 1 h, 3 h, 6 h, and Post 9h_activin ACs) of upregulated DEGs in Post 3h_ACs (4149 genes) using the Algorithm for the Reconstruction of Accurate Cellular Networks (ARACNE)^75,76^ plugin tool in Cytoscape. The mutual information (MI) value was over 0.8 for the inferred *socs3*.S subnetwork.

### Use of published datasets

The dorsal marginal zone (DMZ)-enriched genes were recovered from Kakebeen et al.^23^ with an additional step to indicate the enrichment of DMZ at stage 11: log transformed counts (DMZ)—log transformed counts (VMZ) > 0.5. The DMZ- and ventral marginal zone (VMZ)-enriched genes were referred to Ding et al.^24^. The gene expression datasets in the whole embryos were referred to Session et al.^25^. Smad2/3-associated genes were recovered from stage 10.5 peak datasets in Gupta et al.^45^, and Smad2/3 and FoxhI target genes from Chiu et al.^46^.

## Data availability

RNA-seq data sets reported in this paper were deposited in the NCBI Gene Expression Omnibus (GEO) database (www.ncbi.nlm.nih.gov/geo; accession number is GSE153925).

## Supplementary Information

Supplementary Information 1.

Supplementary Information 2.

Supplementary Information 3.

Supplementary Information 4.

Supplementary Information 5.

Supplementary Information 6.

Supplementary Information 7.

Supplementary Information 8.

Supplementary Information 9.
