# Supplementary material for: Temporal transcriptomic profiling reveals dynamic changes in gene expression of Xenopus animal cap upon activin treatment
Source: Sci Rep. 2021 Jul 15;11:14537. doi: 10.1038/s41598-021-93524-x (PMC8282838; doi:10.1038/s41598-021-93524-x)

Temporal transcriptomic profiling reveals dynamic changes in gene expression of *Xenopus* animal cap upon activin treatment

Yumeko Satou-Kobayashi[1,2,3], Jun-Dal Kim[3,4], Akiyoshi Fukamizu[3] and Makoto Asashima[1,2,3,*]

This PDF file includes:
Supplementary Figs. S1-S10

Supplementary Figure S1. Effects of activin A at different concentrations on the temporal expression of mesoderm-expressing genes.

Total RNA was isolated from ACs immediately after dissection (Pre_activin; sibling embryo reached stages 8.5–9), and from ACs after cultivation in 50 or 10 ng/mL activin A solution for 1, 3, 6, and 9 h (Post 1h_activin, stage 9.5; Post 3h_activin, stages 10–10.25; Post 6h_activin, stages 10.5–11; and Post 9h_activin, stage 11.5, respectively), and used in semiquantitative RT-PCR. Relative expression of the tested genes was determined by dividing the average of the band intensities of three replicates with that of *eef1a1*, which was used as the reference gene. Error bars indicate s.e.m. (n=3). The expression of the dorsal mesoderm genes, *chrd*, *cer1*, and *myf5*, as well as a ventral mesoderm gene, *wnt8a*, was higher in 50 ng/mL activin A-treated ACs than that in 10 ng/mL activin A-treated ACs. The pan-mesodermal marker, *t*, and other dorsal mesoderm genes, *gsc*, and *otx2*, exhibited equivalent expression peaks in ACs treated with 10 and 50 ng/mL activin A.

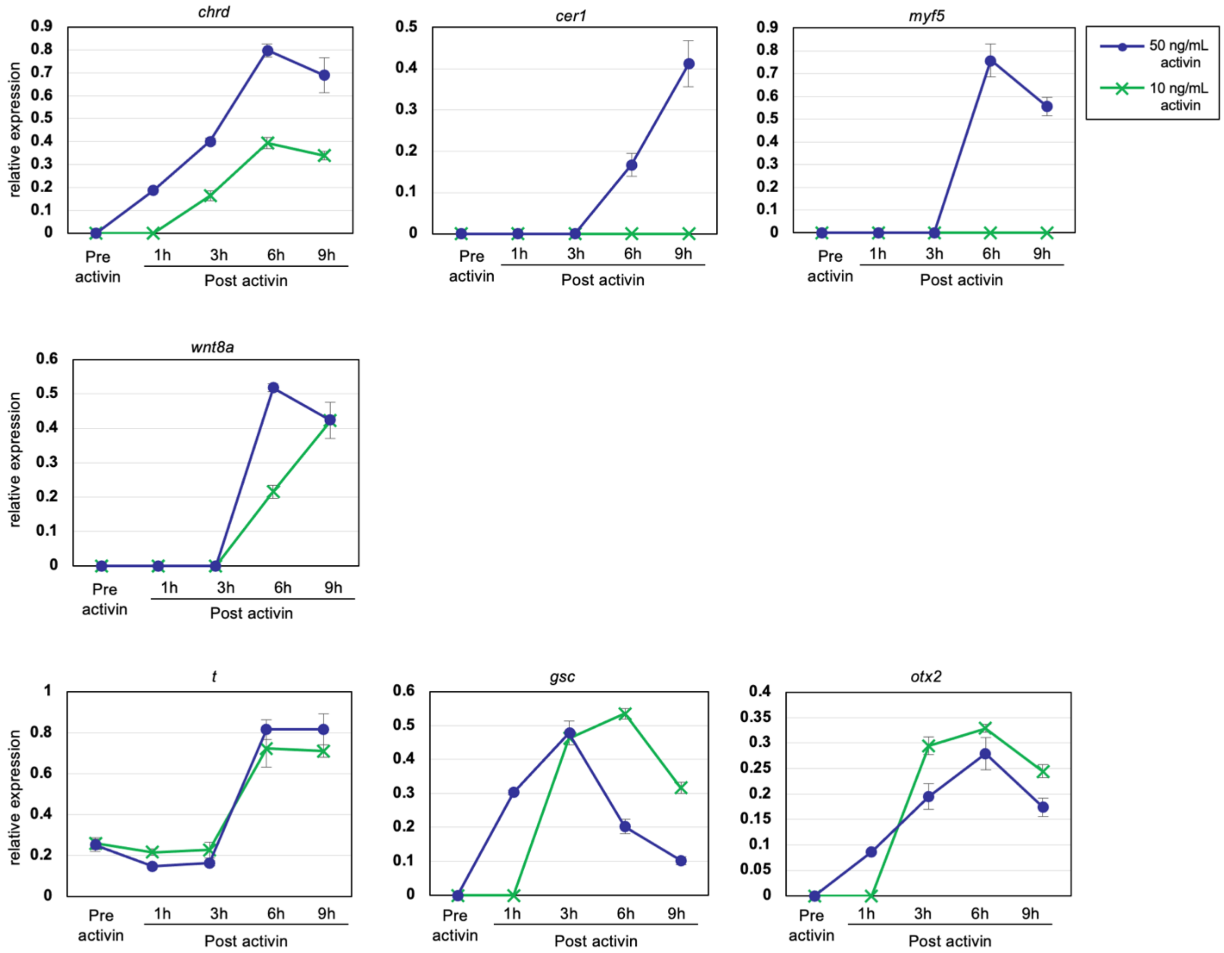

Supplementary Figure S2. Mesoderm-inducing activity of activin A in ACs.

(A) Morphological changes in ACs following activin A treatment. ACs were dissected from blastula and incubated in medium supplemented with (left) or without (right) activin A for 48 h. (B) The expression of notochord marker, *chrd*, and somite markers, *myf5*, *actc1*, and *myogenin*, in ACs following activin A treatment. Total RNA was isolated from ACs immediately after dissection (Pre_activin; stages 8.5-9), and ACs after the cultivation in activin A solution for 6, 9, 12, 24, and 48 h (Post 6h_activin, stages 10.5–11; Post 9h_activin, stage 11.5; Post 12h_activin, stages 15–16; Post 24h_activin, stages 23–24; and Post 48h_activin, stages 32–35, respectively), and were then subjected to semiquantitative RT-PCR. The relative expression of the tested genes was determined by dividing the average of the band intensities of three replicates with that of *eef1a1* as the reference gene. Error bars indicate s.e.m. (n=3).

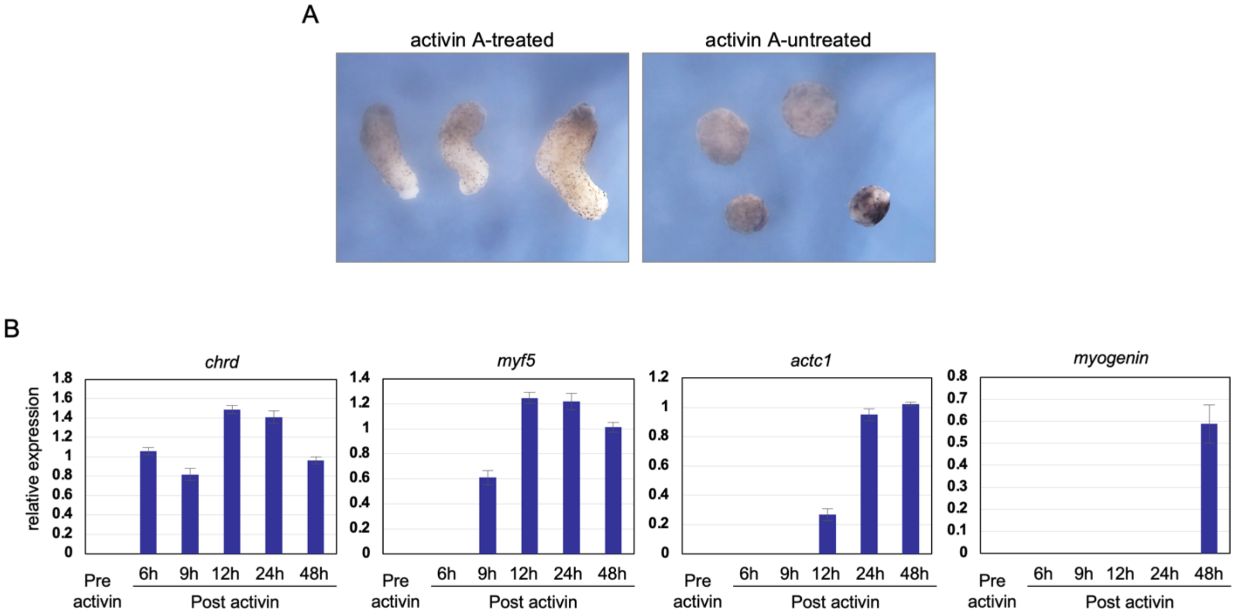

Supplementary Figure S3. Temporal expression of *ccnb1*, *eomes*, *lhx1* and *myf5* determined through semiquantitative RT-PCR and RNA-seq analysis.

(A) Electrophoresis gel of RT-PCR products obtained through RT-PCR. #1-3 means three biological replicates, 1-3. Tested genes are represented, and the probe of eef1a1 was used as loading control. Full size images of each gel were presented in Supplementary Figure S9. (B) Densitometrical analysis for the expression of genes. Relative expression of tested genes was determined by dividing the average of the band intensities of three replicates with that of *eef1a1* as a reference gene. (C) Quantified expression of tested genes examined through RNA-seq. Expression levels of tested genes were calculated by determining the average of the RPKM value of three replicates. *Xenopus laevis* is an allotetraploid species, and contains two set of genes, L genes and S genes. The expression of L and S genes are indicated as colored bar graph: blue, L gene; orange, S gene. (B,C) Error bars indicate s.e.m. (n=3).

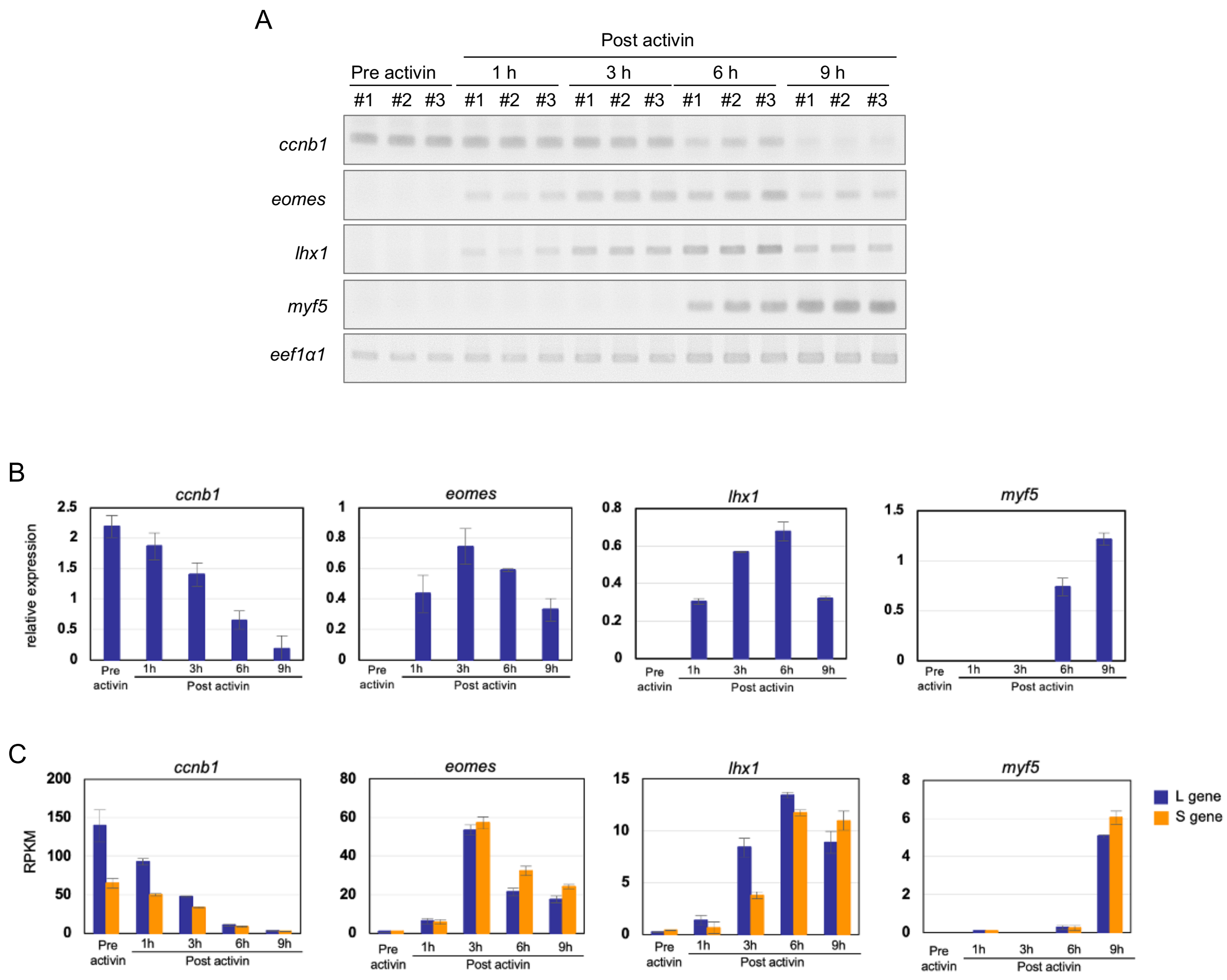

Supplementary Figure S4. Quantified expression of *chrd*, *cer1*, *wnt8a*, *t*, *gsc*, and *otx2* examined through RNA-seq.

Expression levels of the tested genes were calculated by determining the average of the RPKM value of the three replicates. The expression of the L and S genes is indicated on the graph: blue, L gene; orange, S gene. Error bars indicate s.e.m. (n=3).

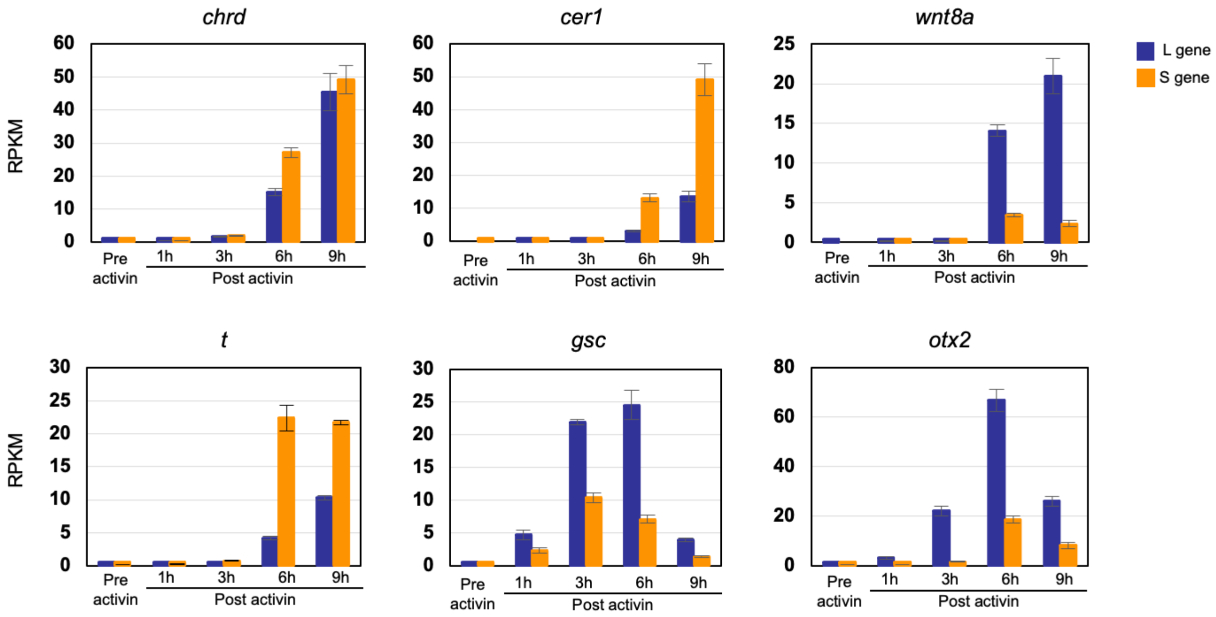

Supplementary Figure S5. Overlap among downregulated DEGs.

DEGs were identified in the comparison of gene expression between Pre_activin and each Post_activin sample based on log2 fold change ≥ 2 or ≤ −2 with FDR at $p<0.05$. The numbers of common and unique downregulated DEGs among Post_activin groups are displayed.

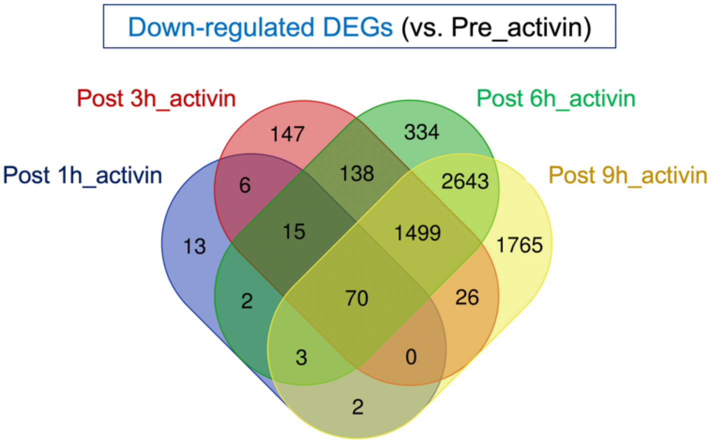

Supplementary Figure S6. ***socs3*** **subnetwork and potential associated genes.**

The inferring *socs3*.S subnetwork was constructed from the gene expression profiles of upregulated DEGs in Post 3h_activin ACs. (A) The first neighboring genes of the *socs3*.S subnetwork. The *socs3*.S subnetwork comprised 75 nodes with 84 edges. (B) The interaction among *socs3*.S, *gata4*.S, *mapk1*.S, and *pou5f3.2*.S. (A,B) *socs3*.S, green ellipse; the potential associated genes, pink and yellow ellipses; black lines, edges.

A

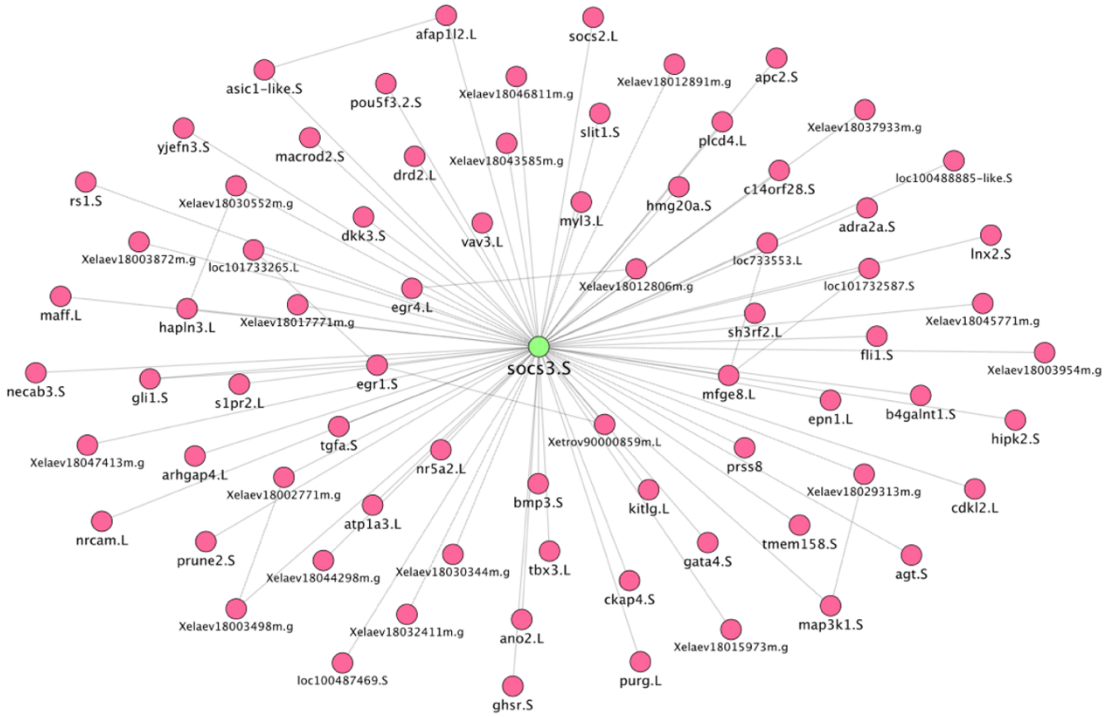

B

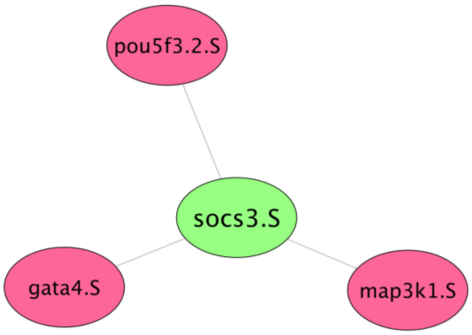

Supplementary Figure S7. Schematic of expressions of organizer and ventral mesoderm expressing genes in the *Xenopus* gastrula.

In the *Xenopus* gastrula (stages 10-10.5), *foxa4*, *eomes*, *gsc*, *lhx1*, *otx2*, *chrd,* and *cer1* genes are expressed in the organizer region. In contrast, *ventx1* and *wnt8* are expressed in the ventral mesoderm. Expression domains of representative genes are illustrated as colored areas of the sagittal section of gastrula, as indicated. Right, dorsal side; left, ventral side.

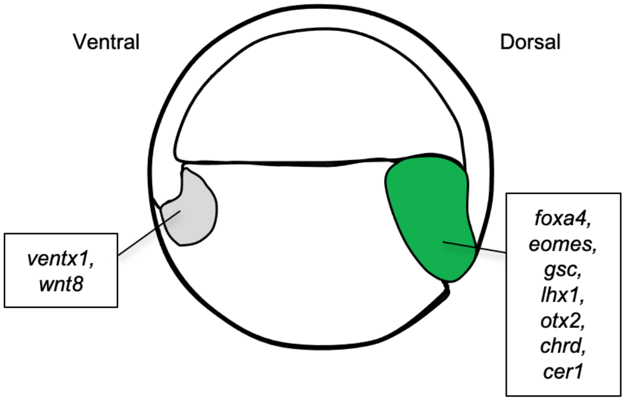

Supplementary Figure S8. Comparison of previously reported Smad2/3-associated genes with our dataset.

Gupta et al. (2014) published the data of a Smad2/3 ChIP-seq analysis, and Chiu et al. (2014) published the data of a Smad2/3 and FoxhI ChIP-seq analysis in *X. tropicalis* gastrula stage embryos (stages 10.5-11). (A) Venn diagram showing the comparison of genes in clusters A-D from our dataset with Smad2/3-associated genes reported in the dataset of Gupta et al. (2014) (left), or with Smad2/3 and FoxhI target genes described in the dataset of Chiu et al. (2014) (right). The number of common and unique genes in independent studies is displayed in colored circles (blue, previous studies; red, this study). (B) The percentage of Smad2/3-associated genes (reported by Gupta et al.) and others in each cluster. (A,B) Our dataset using *X. laevis* contained two copies of genes (L and S genes); therefore, we counted both homeologs when either one overlapped with Smad2/3-associated genes from *X. tropicalis*.

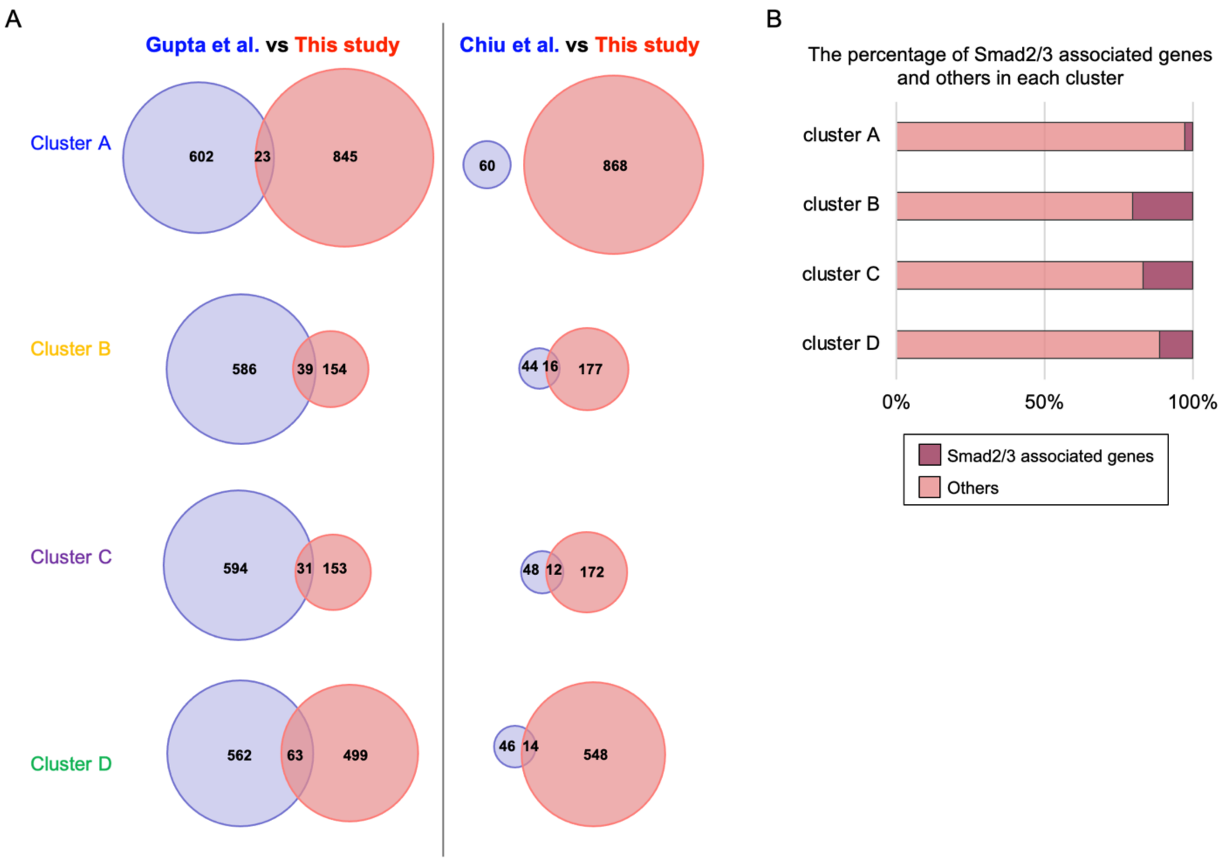

Supplementary Figure S9. Expression of *ccnb1*, *eomes*, *lhx1*, *myf5*, and *eef1a1* evaluated by RT-PCR.

The full electrophoresis gel images of Supplementary Figure S3A. Total RNA was isolated from ACs immediately after dissection (Pre_activin; stages 8.5-9), and ACs after cultivation in activin A solution for 1, 3, 6, and 9 hours (Post 1h_activin; stage 9.5, Post 3h_activin; stages 10-10.25, Post 6h_activin; stages 10.5-11, and Post 9h_activin; stage 11.5), and subjected to semiquantitative RT-PCR. #1-3 means three biological replicates, 1-3. RT-, absence of reverse transcriptase; M, size marker.

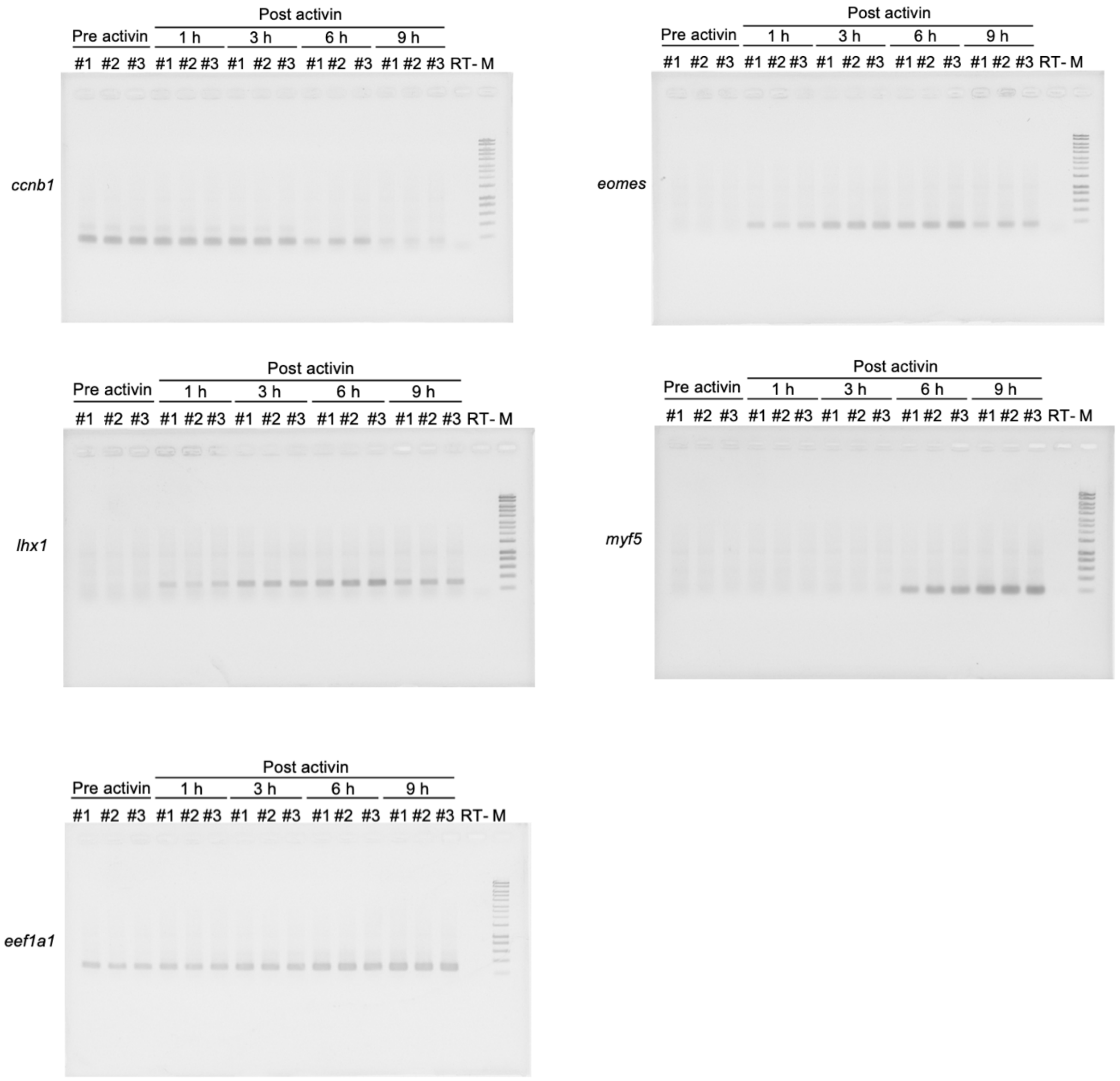

Supplementary Figure S10. Temporal expression of *socs3* and *eef1a1* evaluated by RT-PCR. The full electrophoresis gel images of Figure 5A. Total RNA was isolated from ACs immediately after dissection (Pre_activin; stages 8.5-9), and ACs after culture in the presence (+) or absence (-) of activin A solution for 1, 3, and 9 hours (Post 1h_activin; stage 9.5, Post 3h_activin; stages 10-10.25, and Post 9h_activin; stage 11.5), and subjected to semiquantitative RT-PCR. #1-3 means three biological replicates, 1-3. RT-, absence of reverse transcriptase; M, size marker.

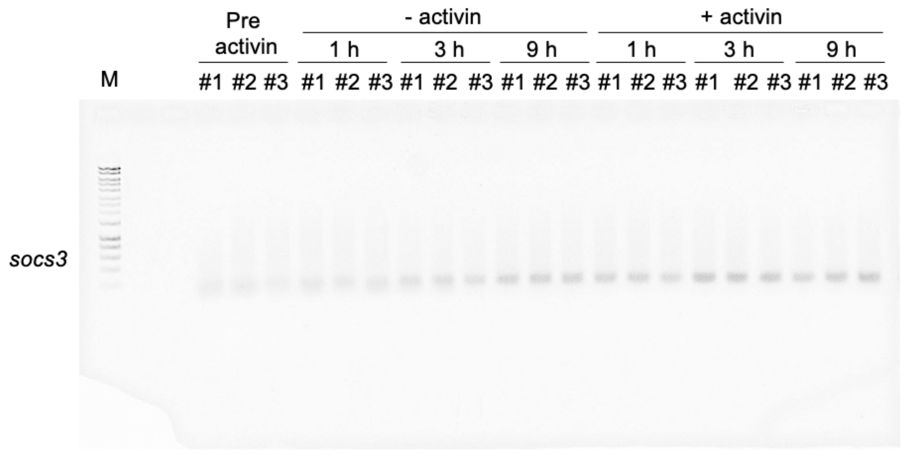

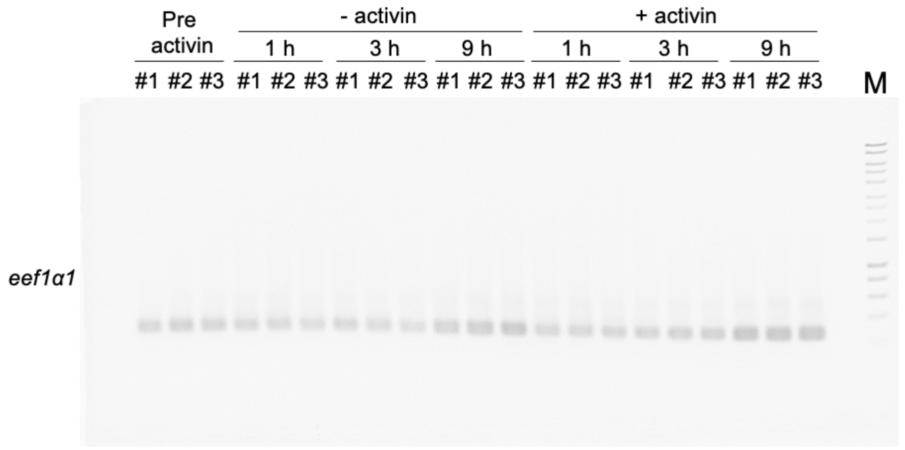

Supplement: Supplementary file 1 — Supplementary Information 1. [file 41598_2021_93524_MOESM1_ESM.pdf]
